# Electrical characteristic fluctuation of 16-nm-gate high-κ/metal gate bulk FinFET devices in the presence of random interface traps

**DOI:** 10.1186/1556-276X-9-633

**Published:** 2014-11-25

**Authors:** Sheng-Chia Hsu, Yiming Li

**Affiliations:** 1 Parallel and Scientific Computing Laboratory, National Chiao Tung University, 1001 Ta-Hsueh Road, Hsinchu 300, Taiwan; 2 Institute of Communications Engineering, National Chiao Tung University, 1001 Ta-Hsueh Road, Hsinchu 300, Taiwan; 3 Department of Electrical and Computer Engineering, National Chiao Tung University, 1001 Ta-Hsueh Road, Hsinchu 300, Taiwan

**Keywords:** Density of interface traps, Random interface traps, Bulk FinFETs, Interface trap fluctuation, Electrical characteristic fluctuation, Statistical device simulation

## Abstract

In this work, we study the impact of random interface traps (RITs) at the interface of SiO_*x*_/Si on the electrical characteristic of 16-nm-gate high-κ/metal gate (HKMG) bulk fin-type field effect transistor (FinFET) devices. Under the same threshold voltage, the effects of RIT position and number on the degradation of electrical characteristics are clarified with respect to different levels of RIT density of state (*D*_it_). The variability of the off-state current (*I*_off_) and drain-induced barrier lowering (DIBL) will be severely affected by RITs with high *D*_it_ varying from 5 × 10^12^ to 5 × 10^13^ eV^−1^ cm^−2^ owing to significant threshold voltage (*V*_th_) fluctuation. The results of this study indicate that if the level of *D*_it_ is lower than 1 × 10^12^ eV^−1^ cm^−2^, the normalized variability of the on-state current, *I*_off_, *V*_th_, DIBL, and subthreshold swing is within 5%.

## Background

For the last decades, the technology of silicon-based CMOS devices suffered significant fabrication challenges and sizeable characteristic variability [1-5]. Characteristics could be affected by various traps in high-κ/metal gate (HKMG) devices [6]. For emerging ultra-scaled transistors, the characteristic degradation induced by interface traps at the interface of SiO_*x*_/Si is severe for giga-scale circuit designs [7]. Furthermore, random interface traps (RITs) appearing at the interface of SiO_*x*_/Si depend on different fabrication processes of HKMG [8-13]. Except planar MOSFETs, fin-type field effect transistors (FinFETs) with HKMG play a key role in sub-22-nm technology nodes to boost electrical performance [14-16] and suppress various fluctuations. Recent studies reported the density of interface traps (*D*_it_) resulting from the orientations of the vertical fin channel of FinFETs [6,17]. The effects of RITs on sub-22-nm FinFETs have also been reported and compared between different device structures [18,19]. Unfortunately, the impact of RITs on 16-nm-gate HKMG bulk FinFET devices has not been clearly discussed yet.

In this work, we study the DC characteristic fluctuation induced by RITs at the SiO_*x*_/Si interface of 16-nm TiN/HfSiON gate stack bulk FinFET devices by using experimentally calibrated three-dimensional (3D) device simulation. Under the same threshold voltage, more than 50% suppressions on the standard deviation of threshold voltage and subthreshold swing (SS) are achieved, benefiting from the nature of the vertical channel, compared with the planar MOSFET devices. By considering different levels of *D*_it_, the effects of RIT position and number on the degradation of electrical characteristics are also examined. This paper is organized as follows: In the ‘Methods’ section, we illustrate the RIT simulation flow. In the ‘Results and discussion’ section, we report the results and discuss the characteristic fluctuation resulting from RITs on 16-nm-gate bulk FinFET devices. Finally, the conclusions are drawn.

## Methods

### RIT simulation method for FinFET devices

**Figure 1:**
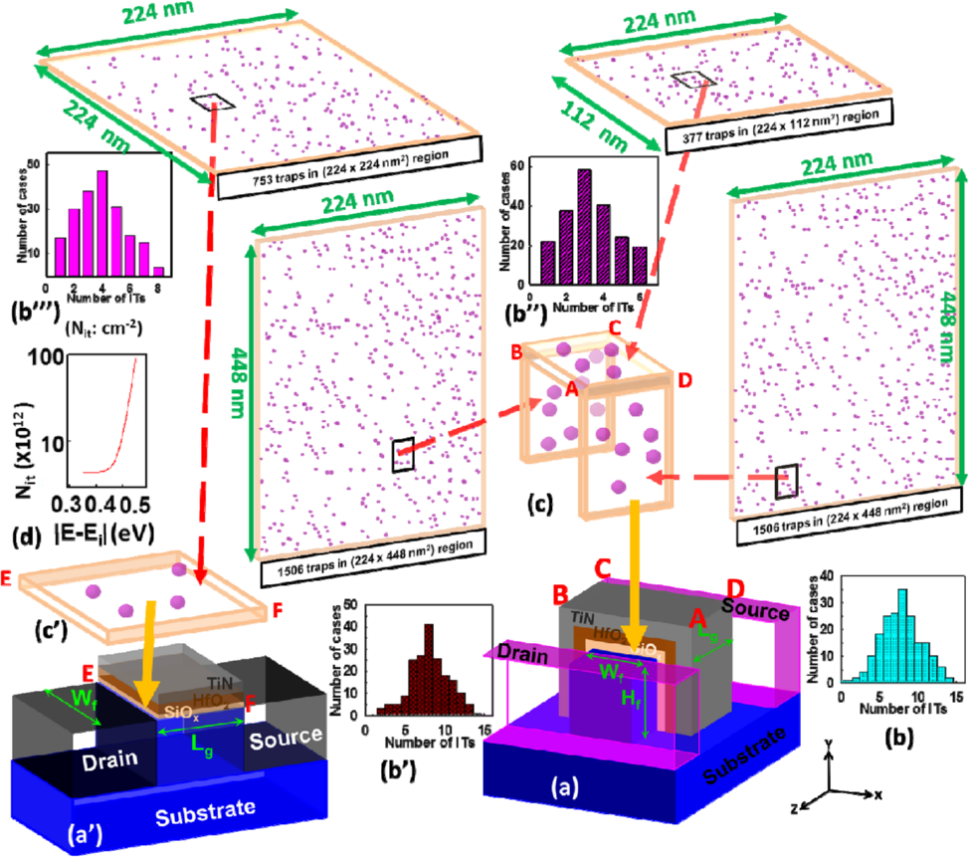
**The RIT simulation technique for the bulk FinFET devices.** (a) and (a’) are the device structures. (b)-(b”’) are the distribution of ITs. (c) and (c’) illustrate the IT planes. (d) shows the individual trap’s density versus the trap’s energy.

We study Si-based 16-nm-gate HKMG bulk FinFETs and planar MOSFET with amorphous-based titanium nitride/hafnium oxide/silicon oxide (TiN/HfO_2_/SiO_*x*_) stacks of gate dielectric and an effective oxide thickness (EOT) of around 0.95 nm (EOT = *T*_o_ + *T*_h_ × *ϵ*_SiO2_/*ϵ*_HfO2_ = 0.6 + 2 × 3.9/22 = 0.95 nm), where *T*_o_ is the thickness of SiO_*x*_, *T*_h_ is the thickness of HfO_2_, and the dielectric constant of HfO_2_ is assumed to be 22. An aspect ratio of 4 (i.e., *H*_f_/*W*_f_ = 32 nm/8 nm = 4), a 30-nm-long source/drain (*S*/*D*), and an 8-nm-long *S*/*D* extension for the explored FinFET are considered, as shown in Figure 1 (a). The doping applied to the channel (*N*_ch_), source/drain (*N*_S/D_), substrate (*N*_B_), and source/drain extension regions is 4 × 10^18^ cm^−3^, 5 × 10^20^ cm^−3^, 10^15^ cm^−3^, and 6 × 10^18^ cm^−3^ for the n-type 16-nm-gate HKMG bulk FinFET devices, respectively. First, we calibrate the nominal DC characteristic of the studied devices according to the International Technology Roadmap for Semiconductor (ITRS) roadmap for low-power applications, which was experimentally quantified in our recent study, and fix the threshold voltage at 300 mV. To estimate device characteristics, a set of 3D drift-diffusion equations coupled with the density gradient equation for quantum correction is performed [20-23]. The mobility model used in the 3D device simulation involves fin channel surface roughness, high-field saturation, and impurity scattering. The mobility model was quantified with our device measurements for the best accuracy, and the characteristic fluctuation was validated with the experimentally measured DC baseband data from 15/20-nm CMOS and FinFET devices in our earlier work [24].

To perform 3D device simulation with two-dimensional (2D) interface trap fluctuation (ITF) for each randomly generated device sample, we assume that the size of each RIT (*S*_RIT_) is equal to 2 nm × 2 nm at the interface of SiO_*x*_/Si. Notably, the value of *S*_RIT_ is numerically set for the simulation of ITF [2,25]. To generate RITs for the statistical device simulation of ITF, we first generate 3,389 acceptor-like traps marked as pink color in the three large 2D planes for the n-type FinFET device, as shown in Figure 1, and the corresponding concentration of RITs is around 1.5 × 10^12^ cm^−2^[26,27]. The total number of generated acceptor-like traps follows the Poisson distribution, as shown in Figure 1 (b)-(b”). Then, we partition the large planes into many subplanes and map them to form a surface of RITs, as shown in Figure 1(c), where the number of traps in the subplanes varies from 0 to 6 at the top side and from 0 to 14 at the lateral sides, and the average number of interface traps is 3, 8, and 8, respectively. The concentration of each RIT (*N*_it_) on a subplane is randomly assigned according to the RIT’s energy following the relationship, as shown in Figure 1 (d). Then, the level of *D*_it_ is the total product of (*N*_it_ × *S*_RIT_) divided by the total area of the SiO_*x*_/Si interface. Ultimately, we repeat this process until all subplanes are assigned. Notably, each subplane with RITs is numerically solved with the quantum mechanically corrected device model, where the RITs appear at the right-hand side of the Poisson equation.

Notably, the *D*_it_ varies with respect to the different process treatments on the TiN/HfSiON gate stacks [9,11,13], so we also consider the impact of three different *D*_it_ levels on device performance degradation. The ranges of high, typical, and low *D*_it_ vary from 5 × 10^12^ to 5 × 10^13^ eV^−1^ cm^−2^, 1 × 10^12^ to 1 × 10^13^ eV^−1^ cm^−2^, and 5 × 10^11^ to 5 × 10^12^ eV^−1^ cm^−2^, respectively. For the p-type devices, we have a similar simulation setting with modification of the acceptor-like traps to donor-like traps. For the planar MOSFET ITF simulation, it follows our recent work, as shown in Figure 1 (a’), (b”’), and (c’), and the details could be found in [7,28].

## Results and discussion

**Figure 2:**
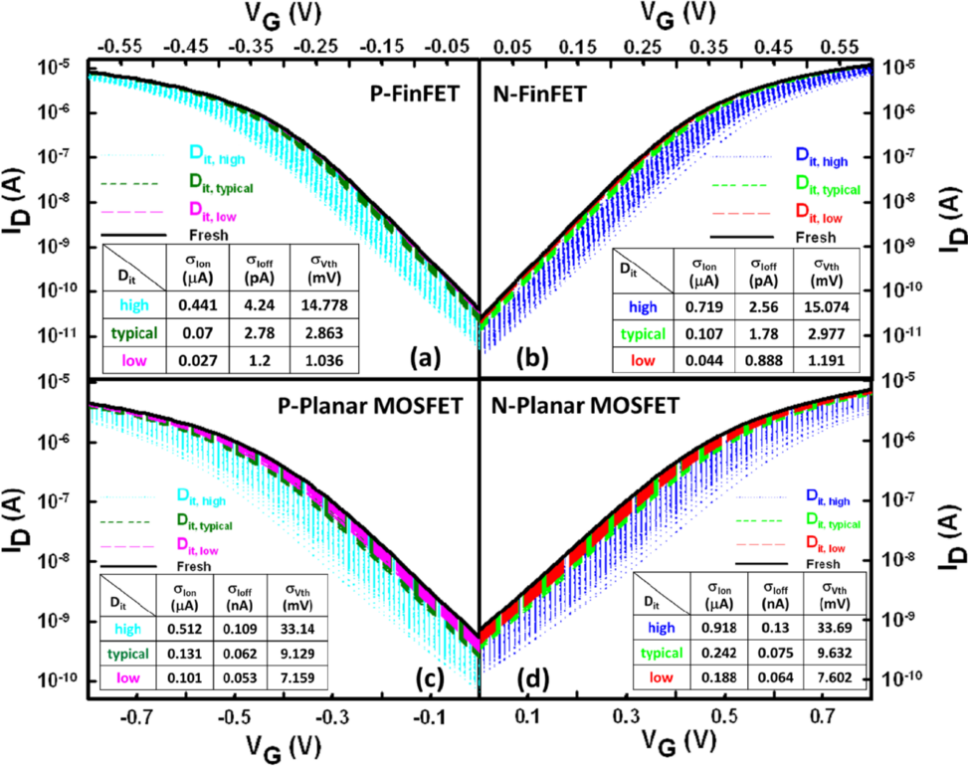
***I***_**D**_**-*V***_**G **_**curves of the studied devices with different *D***_**it **_**levels.** The line indicates the *I*_D_-*V*_G_ of a fresh sample. **(a, b)** n-/p-type bulk FinFET and **(c, d)** n-/p-type planar MOSFET devices.

**Figure 3:**
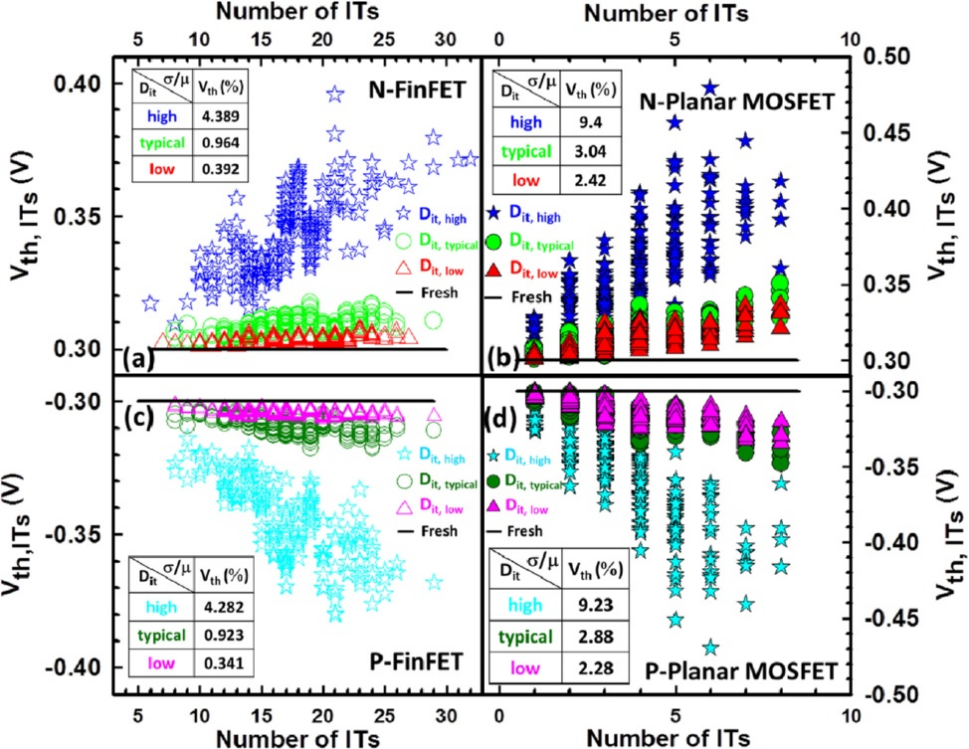
**Fluctuated *V***_**th **_**for the studied devices with different *D***_**it **_**levels.** The line indicates the threshold voltage of a fresh sample. **(a, c)** n-/p-type bulk FinFET and **(b, d)** n-/p-type planar MOSFET devices.

**Figure 4:**
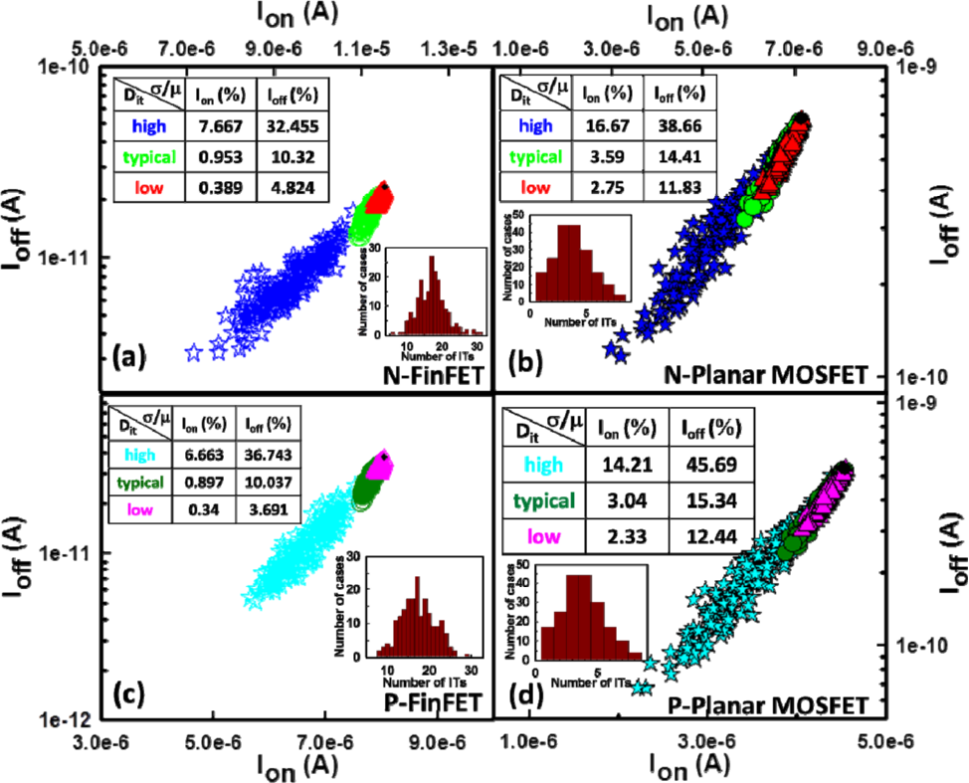
***I***_**off **_**versus *I***_**on **_**for the studied devices with different *D***_**it **_**levels.** The black dot is the result of a free device. **(a, c)** n-/p-type bulk FinFET and **(b, d)** n-/p-type planar MOSFET devices.

The nominal *V*_th_ for the fresh device (i.e., device with ultra-low *D*_it_) is calibrated to 300 mV using a constant-current method. To meet the ITRS roadmap for low-power applications, the voltages applied for the 16-nm-gate HKMG bulk FinFET and planar MOSFET devices were 0.6 and 0.8 V, respectively. As shown in Figure 2, we firstly simulate the RIT-fluctuated *I*_D_-*V*_G_ curves for the n-/p-type bulk FinFET (Figure 2a,b) and n-/p-type planar MOSFET (Figure 2c,d) devices with different levels of *D*_it_, respectively. For all devices, the magnitude of ITF becomes smaller as the level of *D*_it_ decreases. The inset tables list the estimated fluctuation of *I*_on_ (*σI*_on_), *I*_off_ (*σI*_off_), and *V*_th_ (*σV*_th_) for devices with different levels of *D*_it_. As shown in Figure 3, we compare the ITs-fluctuated *V*_th_ under different levels of *D*_it_, where the normalized standard deviation (*σ*/*μ*) of *V*_th_ is calculated, and *σ* and *μ* are the standard deviation and average of the fluctuated cases, respectively. The *V*_th_ shifts and its normalized standard deviation becomes larger when the level of *D*_it_ is increased. Both the n- and p-type FinFET devices, as shown in Figure 3a,c, have comparable magnitudes of *σ*/*μ* which are smaller than that of the planar MOSFET devices (about 50% reduction), as shown in Figure 3b,d. Nevertheless, the ITs-fluctuated *V*_th_ is strongly governed by high *D*_it_ varying from 5 × 10^12^ to 5 × 10^13^ eV^−1^ cm^−2^. In Figure 4, we show the *I*_on_ versus *I*_off_ for all devices with different levels of *D*_it_. The results of the normalized standard deviation of *I*_on_ and *I*_off_ imply that the advantage of the vertical channel in the suppression of ITF will be weakened when the level of *D*_it_ is increased; for example, the ellipsoid-shape distribution of *I*_on_ and *I*_off_ is broadened as the *D*_it_ increases. For the cases of low *D*_it_, as shown in Figure 4a,c, the FinFET *σ*/*μ* of *I*_on_ and *I*_off_ is about three times smaller than that of the planar device, owing to their significant structural dominance. However, such strength is destroyed with the increasing level of *D*_it_; as listed in the inset tables, the normalized standard deviations are considerable and comparable between the two devices for the cases of high *D*_it_, in particular, the *σ*/*μ* of *I*_off_.

**Figure 5:**
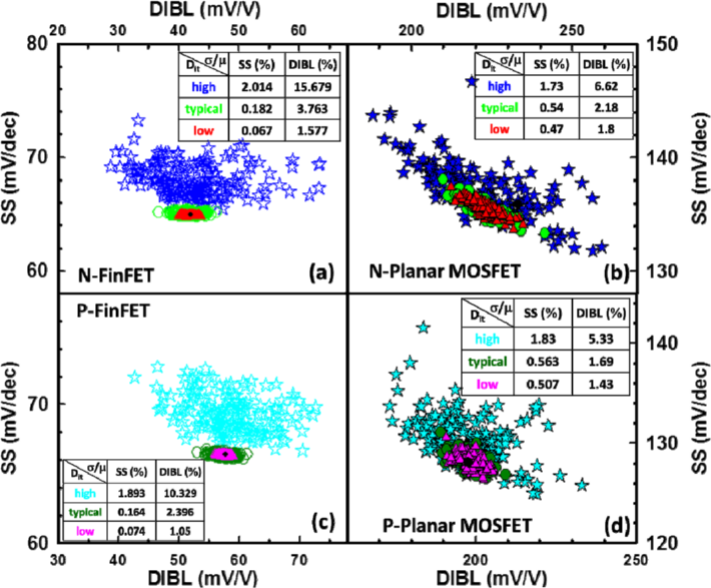
**SS versus DIBL for the studied devices with different *D***_**it **_**levels. (a, c)** n-/p-type bulk FinFET and **(b, d)** n-/p-type planar MOSFET devices.

**Figure 6:**
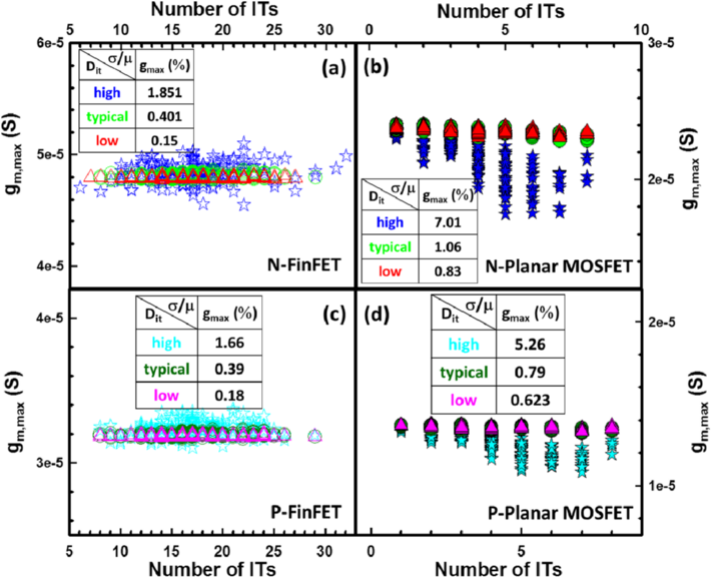
**ITs-fluctuated *g***_**m, max **_**for the studied devices with different *D***_**it **_**levels. (a, c)** n-/p-type bulk FinFET and **(b, d)** n-/p-type planar MOSFET devices.

The degradation of SS becomes more critical when the level of *D*_it_ increases. Owing to large gate capacitance (*C*_g_) coupling in FinFETs, the dependence relationship of ITs-fluctuated SS versus drain-induced barrier lowering (DIBL) is reduced, as shown in Figure 5a,c; however, the distribution of SS versus DIBL exhibits a negative dependency in the planar MOSFETs, as shown in Figure 5b,d. The significant dependence relationship of ITs-fluctuated SS versus DIBL indicates that the characteristic degradation was caused by an even stronger short-channel effect [29]. To maximize *V*_DD_ scaling for logical application, the fluctuations of transconductance (*g*_m_) and subthreshold swing must be minimized. As shown in Figure 6, the ITs-fluctuated transconductances are calculated for the studied devices with different levels of *D*_it_. The flatter normalized standard deviations (within 2%) of the maximum transconductance (*g*_m,max_) listed in the inset tables are found for the FinFET devices with high *D*_it_, as shown in Figure 6a,c.

**Figure 7:**
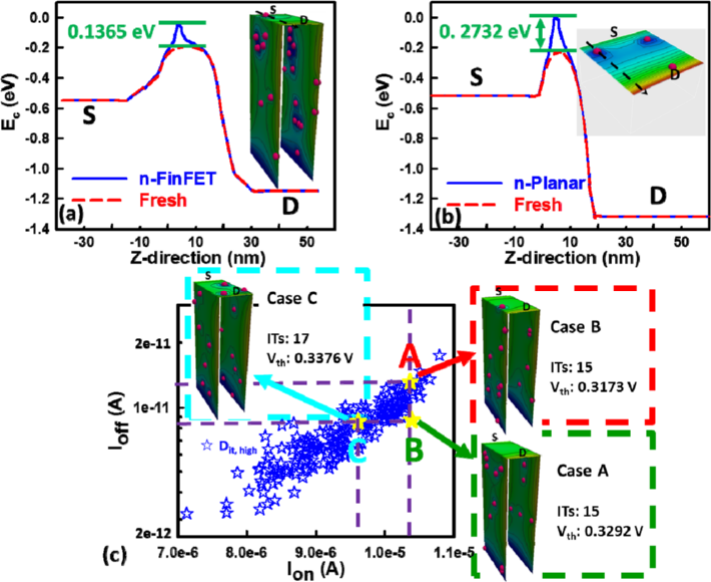
**Barrier height fluctuation induced by RITs for the studied devices with high *D***_**it.**_ n-type HKMG **(a)** bulk FinFET and **(b)** planar MOSFET devices. The devices are under off-state condition, where *V*_G_ = *V*_s_ = *V*_B_ = 0 V and *V*_D_ = 0.6 V for the FinFET and *V*_G_ = *V*_s_ = *V*_B_ = 0 V and *V*_D_ = 0.8 V for the planar MOSFET. **(c)** The *I*_off_-*I*_on_ plot used to explain the position and number effects of RITs.

To go deep into the physics of the results reported above, as shown in Figure 7, we now examine the advantage of the vertical structure and the effect of random distribution (i.e., the random position) and the random number of ITs on the surface potential profiles of the ITs-fluctuated devices. As shown in the inset of Figure 7a, along the channel direction (*Z*-direction) from the source (S) to the drain (D) at the interface of SiO_*x*_/Si on the top gate, the two profiles of conduction band are extracted and compared for the fresh FinFET device and the FinFET device with high *D*_it_ under off-state condition, where the barrier difference induced by a single interface trap is about 0.1365 eV. Similarly, as shown in Figure 7b, for the planar MOSFET device, the barrier difference is about 0.2732 eV. Comparison between Figure 7a and 7b indicates the significant structural effect; the planar MOSFET device severely suffers from the impact of RIT compared to the FinFET one. The coupling of gate electrodes from both the lateral sides to the top gate enhances the *C*_g_, and thus, it effectively reduces the impact of RITs on the energy band. The findings of this comparison confirm the superiority of a 3D channel structure and the aforementioned results. The random position effect of RITs is further examined for the FinFET device. For similar *I*_on_ and different *I*_off_, the two illustration cases (case A and case B) shown in Figure 7c have the same number of ITs (15 ITs) but different *V*_th_ owing to the different positions of RITs. For similar *I*_off_ and different *I*_on_, the numbers of ITs for the two illustration cases (case A and case C) shown Figure 7c are 15 and 17. Therefore, according to the random number effect, they have different *V*_th_ because the effective *D*_it_ of case C is higher than that of case A. Thus, the device has similar *I*_off_ and different *I*_on_.

## Conclusions

In this work, we have investigated the impact of RITs on n-/p-type 16-nm-gate HKMG bulk FinFETs using an experimentally validated device simulation technique. We examined the ITs-fluctuated short-channel effect (SCE) parameters for the bulk FinFET and planar MOSFET devices. Benefiting from the improved gate controllability and stronger gate coupling capability, the estimated normalized standard deviation indicates that the 16-nm-gate HKMG bulk FinFET devices can effectively suppress the DC characteristic and SCE parameter fluctuations induced by RITs with respect to different levels of *D*_it_. The insets of Figures 3, 4, 5, 6 listed the fluctuation magnitudes of *I*_off_ and DIBL which are severely governed by RITs with high *D*_it_ level ranging from 5 × 10^12^ to 5 × 10^13^ eV^−1^ cm^−2^. Due to the strong screening effect for devices under high gate bias, the fluctuation magnitudes of SS and *g*_m_ induced by different levels of RITs are minimized. To effectively control the magnitude of normalized fluctuation within 5% for the *V*_th_, *I*_off_, *I*_on_, SS, and DIBL, the *D*_it_ should be lower than 1 × 10^12^ eV^−1^ cm^−2^. We are currently designing a proper experiment to measure the characteristic fluctuation induced by RITs and study the random bulk traps’ influence together with RITs on device characteristic variability.

## Abbreviations

*σI*_off_: Standard deviation of *I*_off_; *σI*_on_: Standard deviation of *I*_on_; *σV*_th_: Standard deviation of *V*_th_; 2D: Two-dimensional; 3D: Three-dimensional; *C*_g_: Gate capacitance; DIBL: Drain-induced barrier lowering; *D*_it_: Density of interface traps; EOT: Effective oxide thickness; FinFET: Fin-type field effect transistor; *g*_m_: Transconductance; *g*_m,max_: Maximum transconductance; HKMG: High-κ/metal gate; ITs: Interface traps; *I*_off_: Off-state current; *I*_on_: On-state current; *N*_ch_: Channel doping; *N*_B_: Substrate doping; *N*_it_: RIT concentration; *N*_S/D_: Source/drain doping; RITs: Random interface traps; SCE: Short-channel effect; *S*_RIT_: RIT size; SS: Subthreshold swing; *V*_th_: Threshold voltage.

## Competing interests

The authors declare that they have no competing interests.

## Authors’ contributions

S-CH performed the numerical simulation and data analysis. YL conducted the entire study including manuscript preparation. Both authors read and approved the final manuscript.

## References

[B1] Li Y Cheng HW Chiu YY Yiu CY Su HW A unified 3D device simulation of random dopant, interface trap and work function fluctuations on high-κ/metal gate device Proceedings of the IEEE International Electron Devices Meeting 2011 Washington, DC: IEEE 107 110

[B2] Cheng HW Li FH Han MH Yiu CY Yu CH Lee KF Li Y D device simulation of work-function and interface trap fluctuations on high-κ/metal gate devices Proceedings of the International Electron Devices Meeting 2010 San Francisco: IEEE 379 382

[B3] Wang X Brown AR Cheng B Asenov A Statistical variability and reliability in nanoscale FinFETs Proceedings of the International Electron Devices Meeting 2011 Washington, DC: IEEE 103 106

[B4] Penumatcha AV Swandono S Cooper JA Limitations of the high-low C-V technique for MOS interfaces with large time constant dispersion IEEE Trans Electron Devices 2013 60 923 926

[B5] Li Y Cheng HW Chiu YY Interface traps and random dopants induced characteristic fluctuations in emerging MOSFETs Microelectron Eng 2011 88 1269 1271 10.1016/j.mee.2011.03.040

[B6] Tallarico AN Cho M Franco J Ritzenthaler R Togo M Horiguchi N Groeseneken G Crupi F Impact of the substrate orientation on CHC reliability in n-FinFETs—separation of the various contributions IEEE Trans Device Mater Reliab 2014 14 52 56

[B7] Li Y Cheng HW Random interface-traps-induced electrical characteristic fluctuation in 16-nm-gate high-κ/metal gate complementary metal-oxide-semiconductor device and inverter circuit Jpn J Appl Phys 2012 51 04 DC08 10.7567/JJAP.51.04DC08

[B8] Takenaka M Zhang R Takagi S MOS interface engineering for high-mobility Ge CMOS Proceedings of the IEEE International Reliability Physics Symposium 2013 Anaheim, CA: IEEE 4C.1.1 4C.1.8

[B9] Lee JW Simoen E Veloso A Cho MJ Arimura H Boccardi G Ragnarsson LA Chiarella T Horiguchi N Thean A Groeseneken G Low frequency noise analysis for post-treatment of replacement metal gate IEEE Trans Electron Devices 2013 60 2960 2962

[B10] Mao LF Interface traps and quantum size effects on the retention time in nanoscale memory devices Nanoscale Res Lett 2013 8 369 10.1186/1556-276X-8-369 23984827 PMC3847579

[B11] Kapila G Kaczer B Nackaerts A Collaert N Groeseneken GV Direct measurement of top and sidewall interface trap density in SOI FinFETs IEEE Electron Device Lett 2007 28 232 234

[B12] O’Sullivan BJ Hurley PK Leveugle C Das JH Si(100)–SiO_2_ interface properties following rapid thermal processing J Appl Phys 2001 89 3811 3820 10.1063/1.1343897

[B13] Tettamanzi GC Paul A Lee S Mehrotra SR Collaert N Biesemans S Klimeck G Rogge S Interface trap density metrology of state-of-the-art undoped Si n-FinFETs IEEE Electron Device Lett 2011 32 440 442

[B14] Chen SH Liao WS Yang HC Wang SJ Liaw YG Wang H Gu H Wang MC High-performance III-V MOSFET with nano-stacked high-κ gate dielectric and 3D fin-shaped structure Nanoscale Res Lett 2012 7 431 10.1186/1556-276X-7-431 22853458 PMC3466142

[B15] Bijesh R Ok I Baykan M Hobbs C Majhi P Jamm R Datta S Hole mobility enhancement in uniaxially strained SiGe FinFETs: analysis and prospects Proceedings of the IEEE 69th Annual Device Research Conference 2011 Santa Barbara: IEEE 237 238

[B16] Koh SM Samudra GS Yeo YC Contact technology for strained nFinFETs with silicon–carbon source/drain stressors featuring sulfur implant and segregation IEEE Trans Electron Devices 2012 59 1046 1055

[B17] Paul A Tettamanzi GC Lee S Mehrotra SR Collaert N Biesemans S Rogge S Klimeck G Interface trap density metrology from sub-threshold transport in highly scaled undoped Si n-FinFETs J Appl Phys 2011 110 124507 10.1063/1.3660697

[B18] Chen YY Huang WT Hsu SC Chang HT Chen CY Yang CM Chen LW Li Y Statistical device simulation of intrinsic parameter fluctuation in 16-nm-gate n- and p-type bulk FinFETs Proceedings of the IEEE International Conference on Nanotechnology 2013 Beijing: IEEE 442 445

[B19] Wang Y Wei K Liu X Du G Kang J Random interface trap induced fluctuation in 22nm high-k/metal gate junctionless and inversion-mode FinFETs Proceedings of the IEEE International Symposium on VLSI Technology, Systems, and Applications 2013 Hsinchu: IEEE 1 2

[B20] Ancona MG Density-gradient theory: a macroscopic approach to quantum confinement and tunneling in semiconductor devices J Comp Elect 2011 10 65 97 10.1007/s10825-011-0356-9

[B21] Li Y Sze SM Chao TS A practical implementation of parallel dynamic load balancing for adaptive computing in VLSI device simulation Eng Comput 2002 18 124 137 10.1007/s003660200011

[B22] Tang TW Wang X Li Y Discretization scheme for the density-gradient equation and effect of boundary conditions J Comp Elect 2002 1 389 393 10.1023/A:1020764027686

[B23] Odanaka S Multidimensional discretization of the stationary quantum drift-diffusion model for ultrasmall MOSFET structures IEEE Trans Comput Aided Des Integr Circuits Syst 2004 23 837 842 10.1109/TCAD.2004.828128

[B24] Li Y Yu SM Hwang JR Yang FL Discrete dopant fluctuated 20 nm/15 nm-gate planar CMOS IEEE Trans Electron Devices 2008 55 1449 1455

[B25] Andricciola P Tuinhout HP Vries BD Wils NAH Scholten AJ Klaassen DBM Impact of interface states on MOS transistor mismatch Proceedings of the International Electron Devices Meeting 2009 Baltimore: IEEE 711 714

[B26] Cowern NEB Interstitial traps and diffusion in epitaxial silicon films Appl Phys Lett 1994 64 2646 2648 10.1063/1.111479

[B27] Hars G Tass Z Application of quadrupole ion trap for the accurate mass determination of submicron size charged particles J Appl Phys 1995 77 4245 4250 10.1063/1.359480

[B28] Li Y Cheng HW Statistical device simulation of physical and electrical characteristic fluctuations in 16-nm-gate high-κ/metal gate MOSFETs in the presence of random discrete dopants and random interface traps Solid-State Electron 2012 77 12 19

[B29] Mizutani T Kumar A Hiramoto T Analysis of transistor characteristics in distribution tails beyond ±5.4σ of 11 billion transistors Proceedings of the International Electron Devices Meeting 2013 Washington, DC: IEEE 826 829

